# Supplementary material for: Trends in non-COVID-19 hospitalizations prior to and during the COVID-19 pandemic period, United States, 2017–2021
Source: Nat Commun. 2022 Oct 8;13:5930. doi: 10.1038/s41467-022-33686-y (PMC9546751; doi:10.1038/s41467-022-33686-y)
Supplement: Supplementary file 1 — Supplementary Information [file 41467_2022_33686_MOESM1_ESM.pdf]

SUPPLEMENTAL TEXT AND FIGURES

Title: *Trends in non-COVID-19 hospitalizations prior to and during the COVID-19 pandemic period, United States, 2017 – 2021*

Author List: Kelsie Cassell*[1], Casey M. Zipfel[2], Shweta Bansal[2], Daniel M. Weinberger[1]
Affiliations:
[1]Department of Epidemiology of Microbial Diseases, Yale School of Public Health, New Haven CT, USA
[2]Department of Biology, Georgetown University, Washington DC, USA

Supplemental Figure S1.
Number of unique payers in the dataset per month, Jan 1, 2017 – Jun 31, 2021

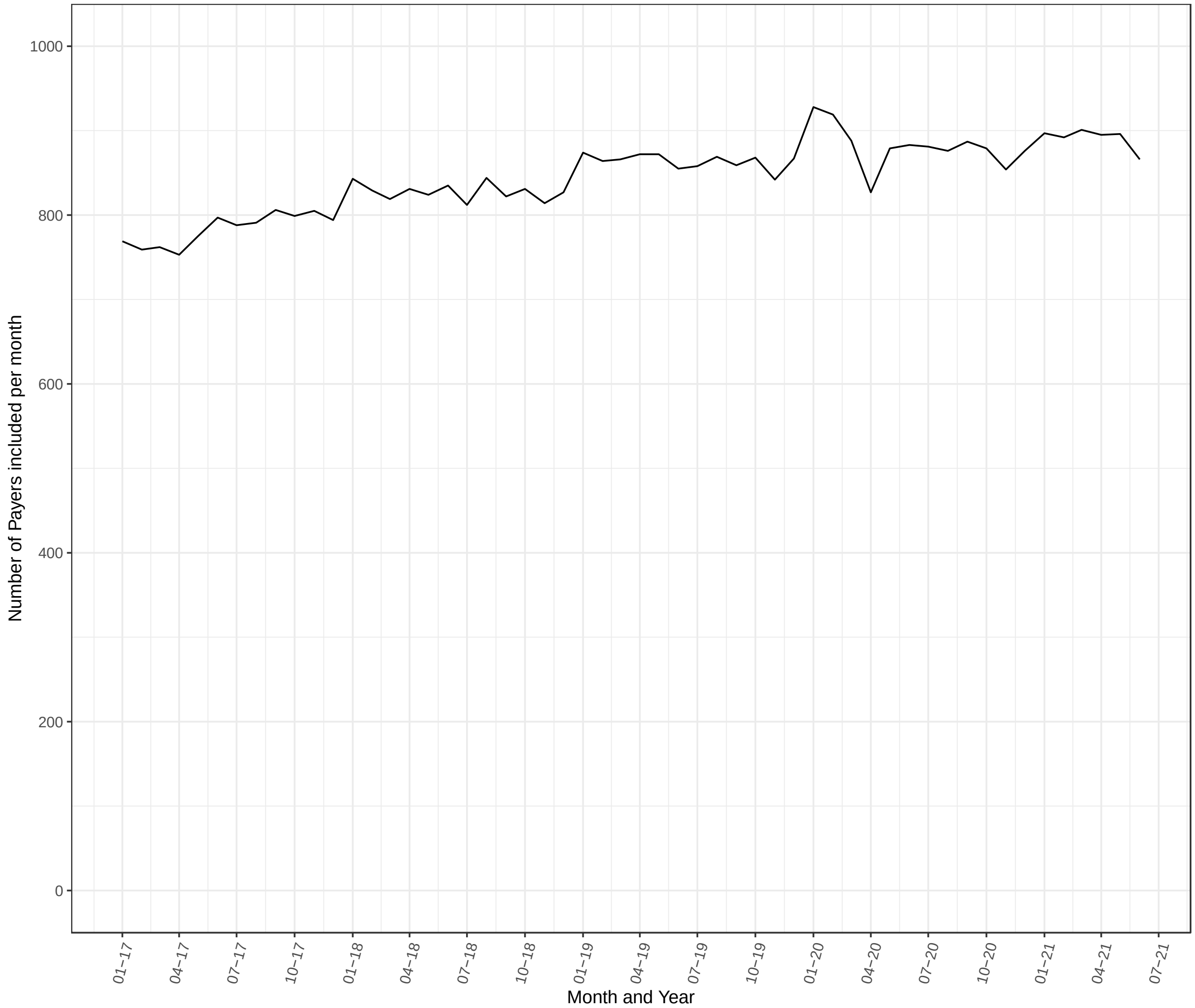

Note: Payers indicate insurance providers, not individual patients hospitalized.

Supplemental Figure S2. Flowchart illustrating the data subsetting process for the COVID-19 Research Database (C19RDB) inpatient data from OfficeAlly, Jan 1, 2017 – Jun 31, 2021

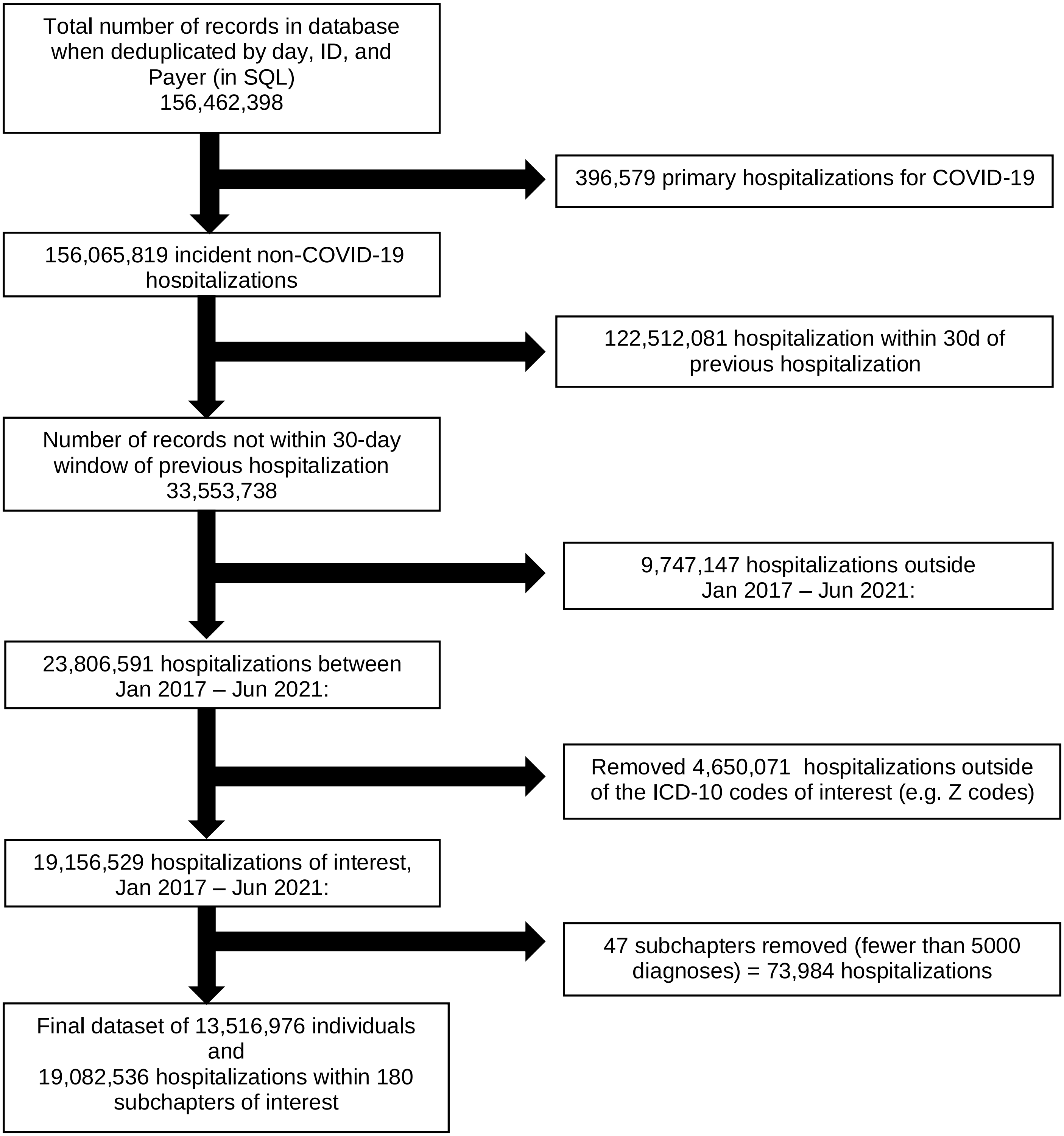

Supplementary Table S1. Categorization of ICD-10 Chapters and diagnostic codes

Chapter No.	ICD-10 Code Range	Description
1	A00 – B99	Certain infectious and parasitic diseases
2	C00 – D49	Neoplasms
3	D50 – D89	Diseases of the blood and blood-forming organs and certain disorders involving the immune mechanism
4	E00 – E89	Endocrine, nutritional and metabolic diseases
5	F01 – F99	Mental, behavioral and neurodevelopmental disorders
6	G00 – G99	Diseases of the nervous system
7	H00 – H59	Diseases of the eye and adnexa
8	H60 – H95	Diseases of the ear and mastoid process
9	I00 – I99	Diseases of the circulatory system
10	J00 – J99	Diseases of the respiratory system
11	K00 – K95	Diseases of the digestive system
12	L00 – L99	Diseases of the skin and subcutaneous tissue
13	M00 – M99	Diseases of the musculoskeletal system and connective tissue
14	N00 – N99	Diseases of genitourinary system
15	O00 – O9A	Pregnancy, childbirth and the puerperium
16	P00 – P96	Certain conditions originating in the perinatal period
17	Q00 – Q99	Congenital malformations, deformations and chromosomal abnormalities
18	R00 – R99	Symptoms, signs, and abnormal clinical laboratory findings, not elsewhere classified
19	S00 – T88	Injury, poisoning and certain other consequences of external causes

Supplementary Table S2.

Excluded subchapters (fewer than 5000 diagnoses between Jan1 2017- June 2021)

Acute rheumatic fever (I00-I02)	Infectious arthropathies (M00-M02)
Arthropod-borne diseases (A90-A99)	Infectious and parasitic sequelae (B90-B94)
Autoinflammatory syndromes (M04)	Inflammatory CNS (G00-G09)
Birth trauma (P10-P15)	Injuries multiple (T07)
Bullous disorders (L10-L14)	Necrotic lower respiratory (J85-J86)
Burns internal (T26-T28)	Neoplasms of bone (C40-C41)
Burns multiple (T30-T32)	Newborn complications (P00-P04)
Certain zoonotic bacterial (A20-A28)	Disorders of optic nerve (H46-H47)
Cleft lip (Q35-Q37)	Other diseases caused by chlamydiae (A70-A74)
Complications of endocrine system (E36)	Other heart disease (I30-I5A)
Complications genitourinary (N99)	Other human herpesviruses (B10)
Complications puerperium (O85-O92)	Other infectious diseases (B99)
Complications trauma (T79)	Other musculoskeletal (M95)
Complications skin (L76)	Other problems with newborn (P84)
Complications spleen (D78)	Other spirochetal diseases (A65-A69)
Complications respiratory (J95)	Periprosthetic fracture (M97)
Congenital malformations respiratory (Q30-Q34)	Diseases of peritoneum (K65-K68)
Digestive disorders of the newborn (P76-P78)	Protozoal disease (B50-B64)
Disorders of glucose and pancreas (E15-E16)	Respiratory-cardio perinatal (P19-P29)
Ear complications (H95)	Rickettsioses (A75-A79)
Endocrine and metabolic disorders of the newborn (P70-P74)	Conditions related to integument and temperature regulation of newborn (P80-P83)
Eye complications (H59)	Tuberculosis (A15-A19)
Frostbite (T33-T34)	Unknown mortality (R99)
Glomerular diseases (N00-N08)	Unspecified mental (F99)
Helminthiases (B65-B83)	Viral prion, infections of CNS (A80-A89)
	Ill-defined and unknown mortality (R99)

Supplemental Figure S3.
Number of non-COVID-19 hospitalizations (in ICD-10 chapters A-T) and COVID-19 hospitalizations (all diagnostic field codes) analyzed, Jan 1, 2017 – Jun 31, 2021

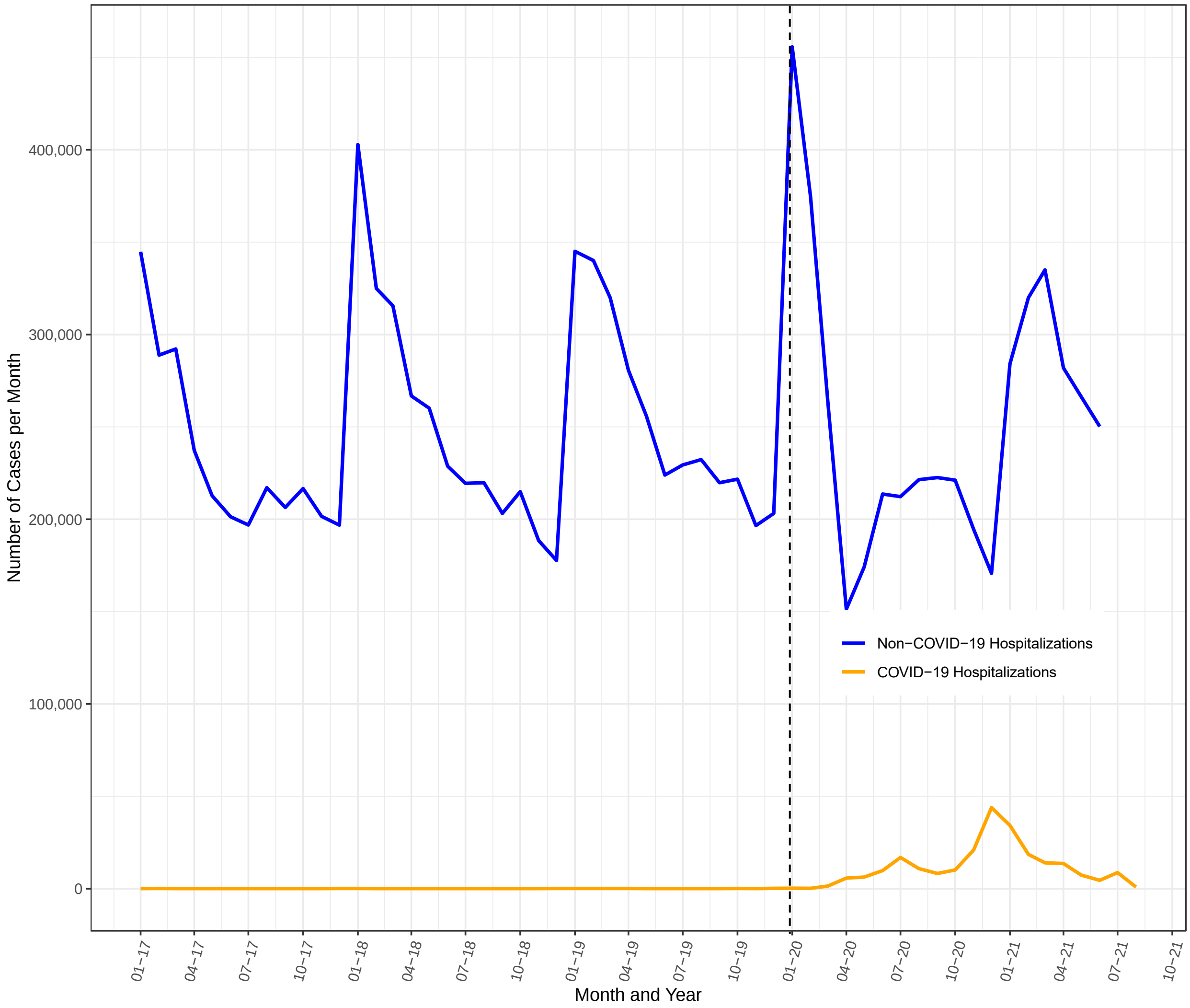

Supplementary Table S3. Estimated IRR and 95% Prediction Interval of select ICD10 chapters between Jan and Apr 2020

	Jan 2020<br>IRR (95%<br>Prediction Interval)	Feb 2020<br>IRR (95%<br>Prediction Interval)	Mar 2020<br>IRR (95%<br>Prediction Interval)	Apr 2020<br>IRR (95%<br>Prediction Interval)
Blood and Blood forming organs<br>(D50-D89)	1.21 (1.17, 1.26)	1.03 (1.00, 007)	0.81 (0.78, 0.84)	0.70 (0.67, 0.72)
Circulatory System<br>(I00-I99)	1.13 (1.11, 1.15)	1.02 (1.01, 1.04)	0.74 (0.72, 0.75)	0.77 (0.76, 0.78)
Congenital malformations, deformations and chromosomal abnormalities<br>(Q00-Q99)	1.11 (1.06, 1.17)	0.96 (0.91, 1.01)	0.64 (0.61, 0.67)	0.45 (0.43, 0.47)
Digestive System<br>(K00-K95)	1.08 (1.07, 1.10)	0.99 (0.98, 1.01)	0.76 (0.75, 0.77)	0.48 (0.47, 0.49)
Ear and Mastoid<br>(H60-H95)	1.08 (1.05, 1.10)	1.02 (1.00, 1.05)	0.67 (0.67, 0.67)	0.28 (0.27, 0.29)
Endocrine, Nutritional, and Metabolic<br>(E00-E89)	1.16 (1.15, .18)	1.05 (1.03, 1.06)	0.79 (0.77, 0.80)	0.69 (0.68, 0.70)
Eye and Adnexa<br>(H00-H59)	1.15 (1.12, 1.18)	1.03 (1.01, 1.06)	0.70 (0.69, 0.72)	0.28 (0.28, 0.29)
Genitourinary System<br>(N00-N99)	1.13 (1.12, 1.15)	0.98 (0.97, 0.99)	0.76 (0.75, 0.77)	0.62 (0.62, 0.63)
Infectious & Parasitic Diseases<br>(A00-B99)	1.13 (1.11, 1.16)	1.07 (1.05, 1.10)	0.95 (0.93, 0.96)	0.61 (0.60, 0.62)
Injury, poisoning, external causes<br>(S00-T88)	1.06 (1.05, 1.07)	1.03 (1.02, 1.04)	0.79 (0.78, 0.79)	0.57 (0.57, 0.58)
Mental, Behavioral, and Neurodevelopmental<br>(F01-F99)	1.16 (1.14, 1.17)	1.03 (1.01, 1.04)	0.85 (0.84, 0.86)	0.91 (0.89, 0.92)
Musculoskeletal system and Connective tissue<br>(M00-M99)	1.12 (1.11, 1.13)	1.00 (0.99, 1.01)	0.71 (0.70, 0.72)	0.45 (0.45, 0.45)
Neoplasms<br>(C00-D49)	1.17 (1.14, 1.19)	1.00 (0.98, 1.02)	0.81 (0.79, 0.83)	0.65 (0.64, 0.67)
Nervous System<br>(G00-G99)	1.16 (1.14, 1.18)	1.07 (1.05, 1.09)	0.71 (0.70, 0.72)	0.68 (0.66, 0.69)
Conditions in the perinatal period<br>(P00-P96)	0.96 (0.87, 1.06)	0.84 (0.76, 0.94)	0.85 (0.77, 0.94)	0.85 (0.77, 0.95)
Pregnancy, Childbirth, and Puerperium<br>(O00-O9A)	1.07 (1.04, 1.09)	0.97 (0.95, 0.99)	0.92 (0.90, 0.94)	0.89 (0.87, 0.91)
Respiratory System<br>(J00-J99)	1.15 (1.14, 1.16)	1.09 (1.08, 1.10)	0.93 (0.92, 0.94)	0.40 (0.40, 0.41)
Signs and Symptoms not elsewhere classified<br>(R00-R99)	1.12 (1.10, 1.13)	1.02 (1.01, 1.03)	0.83 (0.83, 0.84)	0.58 (0.58, 0.58)
Skin and Subcutaneous<br>(L00-L99)	1.10 (1.07, 1.12)	1.01 (1.00, 1.03)	0.77 (0.75, 0.78)	0.60 (0.59, 0.61)

Supplementary Figure S4.
Incidence Rate Ratio (IRR) per Diagnostic Chapter for the months between January 2020 and June 2021S

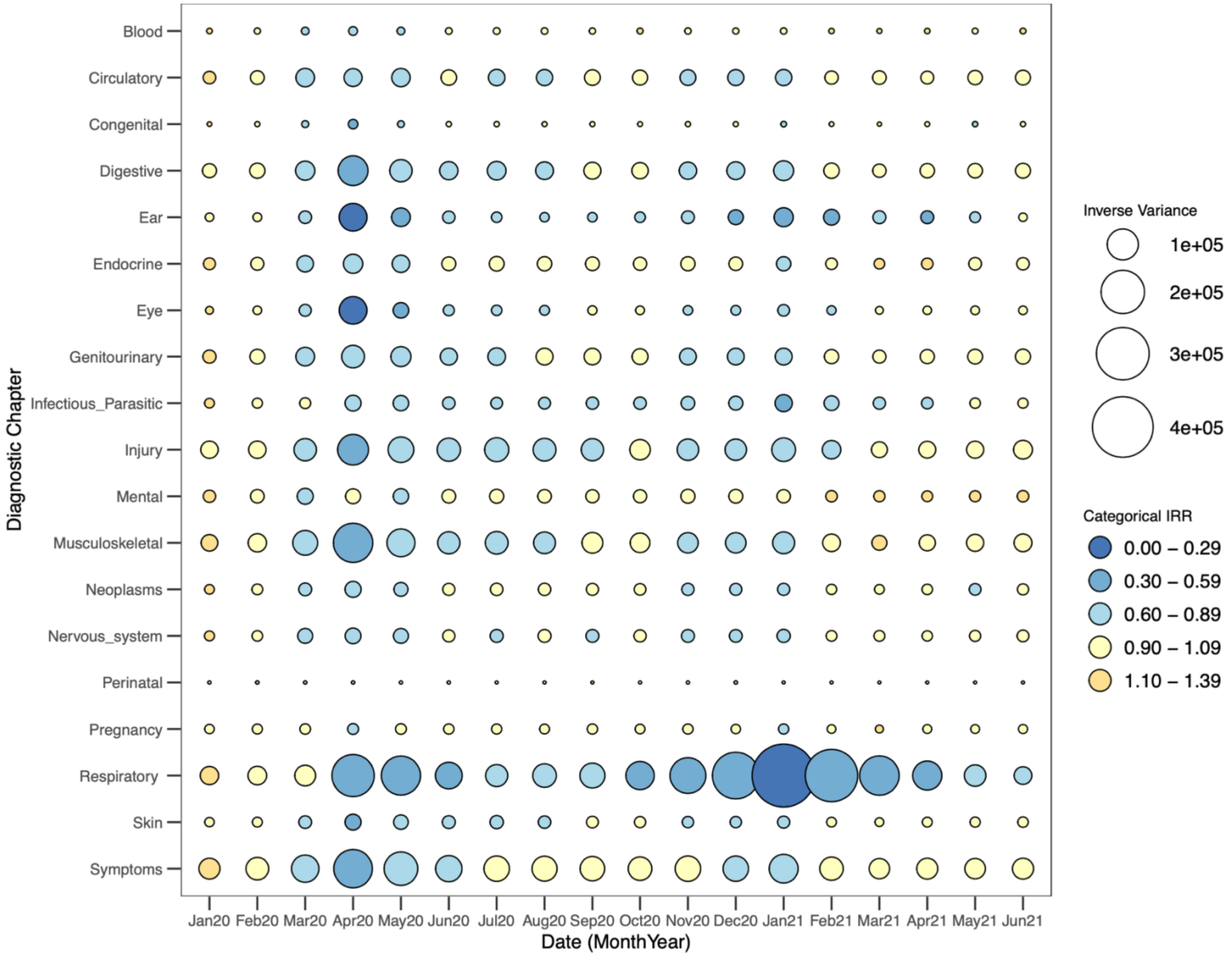

Figure S4 Note: Each bubble is colored according to IRR and the diameter of the bubble is proportional to the inverse of the IRR variance estimate (i.e. a larger bubble signifies greater confidence in the estimate).

Supplemental Figure S5:
Results of hierarchical clustering of subchapters revealing three clusters

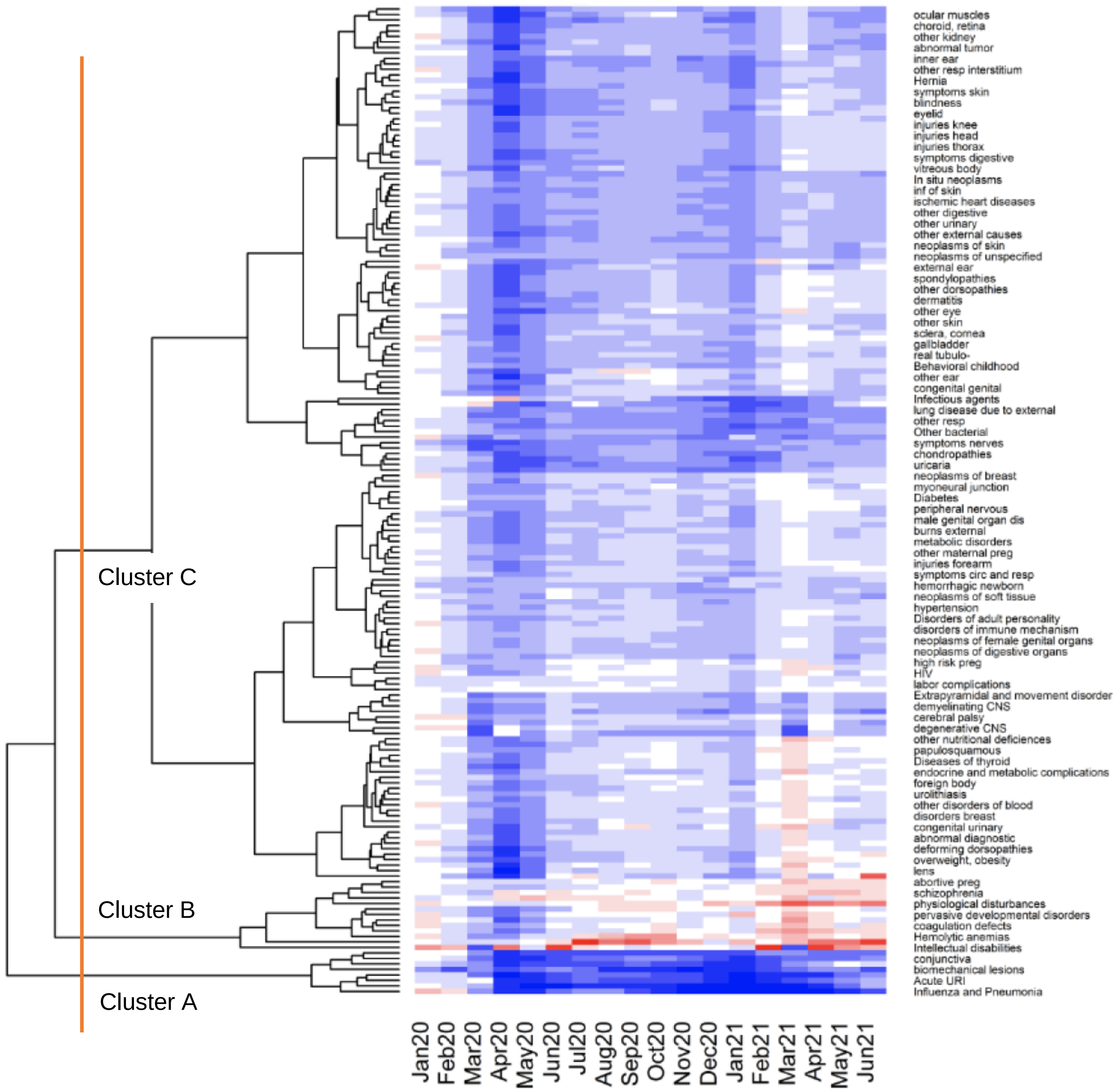

Supplemental Figure S6.
Time series of observed hospitalizations per diagnostic subchapter by month and year (solid line) with model predicted case counts (dashed line) and confidence intervals (grey).

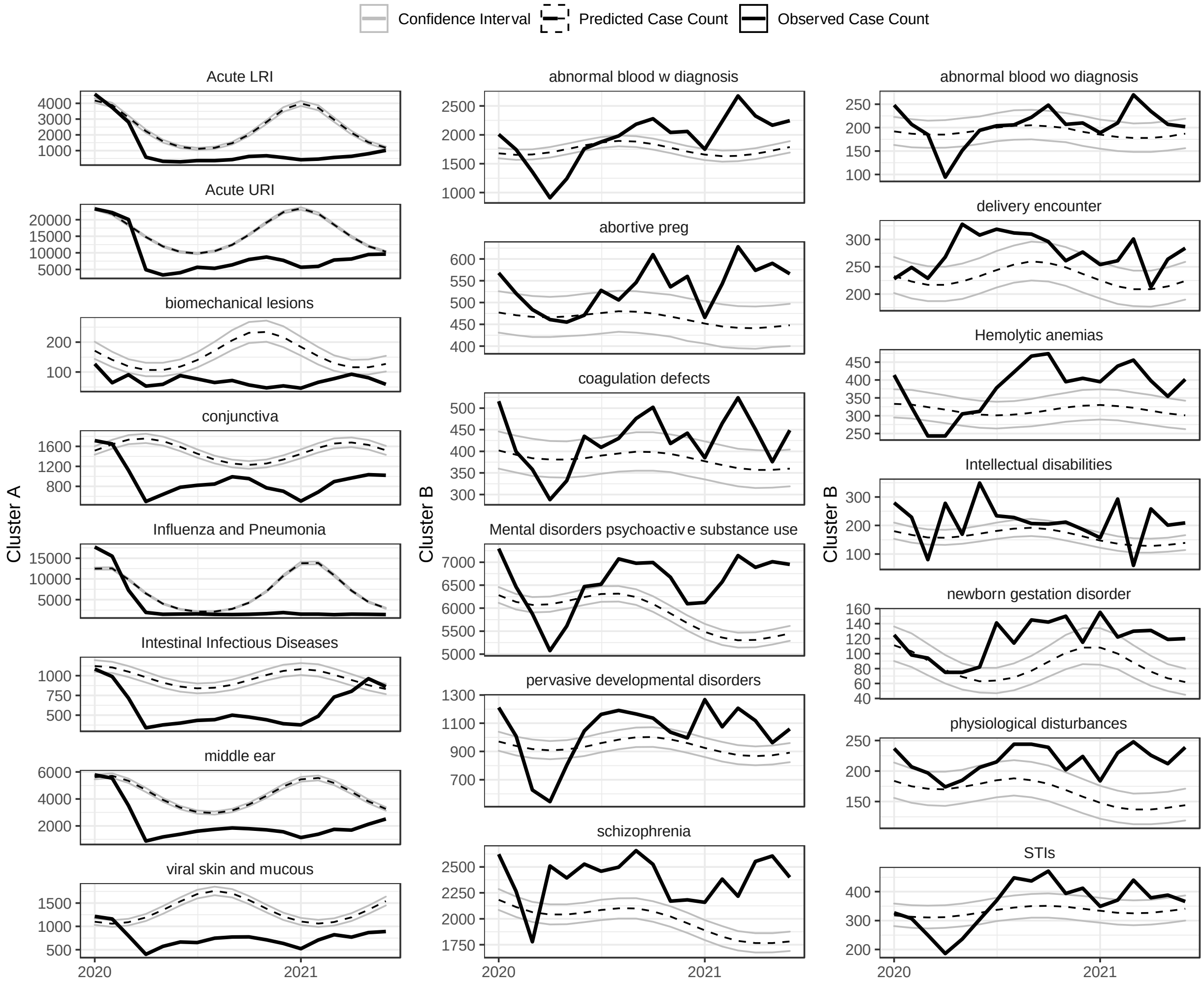

Description Supplemental Figure S6. Data are presented as the observed case count per month (black), with regression model predicted case counts (dashed black) surrounded by a gray prediction interval estimated through two-stage simulation using Monte Carlo resampling that accounted for parameter uncertainty and observation uncertainty

Supplementary Table S4. Incidence Rate Ratio and 95% Prediction interval of select Subchapters of disease, March 2020 to December 2020

Subchapter	Mar	Apr	May	Jun	Jul	Aug	Sept	Oct	Nov	Dec
I&P (J09-J18)	0.74 (0.72, 0.76)	0.29 (0.28, 0.30)	0.37 (0.35, 0.38)	0.58 (0.56, 0.61)	0.74 (0.71, 0.78)	0.68 (0.65, 0.71)	0.51 (0.49, 0.53)	0.35 (0.34, 0.36)	0.23 (0.23, 0.24)	0.17 (0.17, 0.18)
Acute URI (J00 – J06)	1.09 (1.07, 1.08)	0.33 (0.33, 0.34)	0.28 (0.27, 0.28)	0.39 (0.38, 0.40)	0.58 (0.53, 0.59)	0.51 (0.50, 0.52)	0.51 (0.50, 0.52)	0.52 (0.51, 0.53)	0.46 (0.45, 0.46)	0.35 (0.34, 0.35)
Acute LRI (J20-J22)	0.91 (0.87, 0.95)	0.26 (0.25, 0.27)	0.20 (0.19. 0.21)	0.24 (0.22, 0.25)	0.33 (0.31, 0.35)	0.31 (0.29, 0.33)	0.29 (0.27, 0.31)	0.31 (0.30, 0.33)	0.24 (0.23, 0.25)	0.16 (0.15, 0.16)
Viral skin and mucous (B00 – B09)	0.74 (0.69, 0.79)	0.34 (0.32, 0.36)	0.42 (0.40, 0.45)	0.43 (0.41, 0.46)	0.39 (0.37, 0.41)	0.43 (0.40, 0.45)	0.45 (0.43, 0.48)	0.50 (0.47, 0.53)	0.52 (0.49, 0.55)	0.52 (0.49, 0.56)
Middle ear (H65 – H75)	0.66 (0.64, 0.68)	0.19 (0.18, 0.19)	0.30 (0.29, 0.31)	0.41 (0.40, 0.43)	0.53 (0.51, 0.55)	0.59 (0.57, 0.61)	0.59 (0.57, 0.61)	0.50 (0.48, 0.52)	0.40 (0.39, 0.42)	0.32 (0.31, 0.33)
Intestinal Infectious (A00-A09)	0.68 (0.64, 0.73)	0.35 (0.32, 0.37)	0.42 (0.39, 0.44)	0.46 (0.43, 0.50)	0.52 (0.48, 0.56)	0.52 (0.49, 0.56)	0.56 (0.53, 0.61)	0.50 (0.47, 0.54)	0.44 (0.41, 0.47)	0.37 (0.34, 0.40)
Intellectual Disabilities (F70 – F79)	0.51 (0.43, 0.60)	1.77 (1.50, 2.11)	1.04 (0.89, 1.24)	2.04 (1.75, 2.42)	1.30 (1.11, 1.53)	1.21 (1.04, 1.43)	1.08 (0.93, 1.27)	1.10 (0.94, 1.29)	1.20 (1.03, 1.44)	1.15 (0.98, 1.38)
Schizophrenia (F20 – F29)	0.86 (0.82, 0.90)	1.23 (1.17, 1.29)	1.17 (1.12, 1.23)	1.23 (1.17, 1.28)	1.18 (1.13, 1.24)	1.19 (1.14, 1.25)	1.27 (1.21, 1.33)	1.22 (1.16, 1.28)	1.07 (1.02, 1.13)	1.11 (1.06, 1.17)
STI (A50 – A64)	0.80 (0.71, 0.92)	0.60 (0.53, 0.68)	0.74 (0.66, 0.84)	0.93 (0.83, 1.06)	1.10 (0.97, 1.24)	1.30 (1.16, 1.47)	1.25 (1.11, 1.41)	1.34 (1.20, 1.52)	1.13 (1.01, 1.29)	1.21 (1.07, 1.38)
Hemolytic anemias (D55 – D59)	0.75 (0.67, 0.85)	0.77 (0.68, 0.87)	0.99 (0.88, 1.12)	1.03 (0.91, 1.17)	1.26 (1.12, 1.43)	1.39 (1.24, 1.58)	1.52 (1.35, 1.73)	1.50 (1.33, 1.72)	1.22 (1.09, 1.40)	1.23 (1.09, 1.41)
Coagulation defects (D65 – D69)	0.93 (0.83, 1.04)	0.76 (0.68, 0.85)	0.87 (0.78, 0.98)	1.13 (1.02, 1.27)	1.05 (0.95, 1.18)	1.09 (0.98, 1.22)	1.19 (1.07, 1.34)	1.26 (1.13, 1.41)	1.06 (0.95, 1.19)	1.15 (1.02, 1.29)

Supplementary Table S5.
Sensitivity Analysis Results: Incidence Rate Ratio (Median) and 95% sample interval of ICD-10 Chapters of disease, March 2021 to December 2021

	Jan 2020 IRR (95% Prediction Interval)	Feb 2020 IRR (95% Prediction Interval)	Mar 2020 IRR (95% Prediction Interval)	Apr 2020 IRR (95% Prediction Interval)
Blood and Blood forming organs (D50-D89)	1.21 (1.18, 1.26)	1.03 (1.00, 1.07)	0.81 (0.79, 0.84)	0.70 (0.67, 0.73)
Circulatory System (I00-I99)	1.14 (1.10, 1.19)	1.03 (0.99, 1.07)	0.74 (0.72, 0.76)	0.77 (0.71, 0.82)
Congenital malformations, deformations and chromosomal abnormalities (Q00-Q99)	1.11 (1.06, 1.18)	0.96 (0.90, 1.02)	0.64 (0.60, 0.68)	0.44 (0.42, 0.49)
Digestive System (K00-K95)	1.08 (1.06, 1.12)	1.00 (0.98, 1.04)	0.77 (0.75, 0.79)	0.48 (0.46, 0.50)
Ear and Mastoid (H60-H95)	1.08 (1.02, 1.14)	1.02 (0.97, 1.08)	0.67 (0.64, 0.71)	0.28 (0.25, 0.31)
Endocrine, Nutritional, and Metabolic (E00-E89)	1.17 (1.13, 1.21)	1.05 (1.01, 1.08)	0.79 (0.76, 0.81)	0.68 (0.64, 0.72)
Eye and Adnexa (H00-H59)	1.16 (1.11, 1.21)	1.04 (1.00, 1.09)	0.71 (0.68, 0.75)	0.28 (0.26, 0.31)
Genitourinary System (N00-N99)	1.14 (1.09, 1.19)	0.99 (0.96, 1.02)	0.77 (0.74, 0.80)	0.63 (0.60, 0.66)
Infectious & Parasitic Diseases (A00-B99)	1.14 (1.10, 1.18)	1.08 (1.04, 1.12)	0.95 (0.92, 0.99)	0.61 (0.57, 0.67)
Injury, poisoning, external causes (S00-T88)	1.06 (1.03, 1.11)	1.03 (1.00, 1.08)	0.80 (0.77, 0.82)	0.58 (0.56, 0.61)
Mental, Behavioral, and Neurodevelopmental (F01-F99)	1.15 (1.12, 1.19)	1.02 (1.00, 1.05)	0.85 (0.80, 0.87)	0.90 (0.84, 0.94)
Musculoskeletal system and Connective tissue (M00-M99)	1.13 (1.09, 1.17)	1.00 (0.97, 1.04)	0.72 (0.70, 0.74)	0.45 (0.43, 0.47)
Neoplasms (C00-D49)	1.17 (1.14, 1.23)	1.01 (0.98, 1.06)	0.81 (0.79, 0.86)	0.65 (0.63, 0.69)
Nervous System (G00-G99)	1.16 (1.12, 1.21)	1.08 (1.04, 1.12)	0.71 (0.69, 0.73)	0.68 (0.63, 0.73)
Conditions in the perinatal period (P00-P96)	1.00 (0.94, 1.05)	0.86 (0.82, 0.90)	0.88 (0.83, 0.95)	0.87 (0.82, 0.93)
Pregnancy, Childbirth, and Puerperium (O00-O9A)	1.07 (1.03, 1.12)	0.97 (0.94, 1.02)	0.92 (0.89, 0.96)	0.89 (0.86, 0.93)
Respiratory System (J00-J99)	1.15 (1.10, 1.20)	1.09 (1.05, 1.14)	0.93 (0.90, 0.97)	0.41 (0.38, 0.44)
Signs and Symptoms not elsewhere classified (R00-R99)	1.12 (1.09, 1.17)	1.02 (0.99, 1.07)	0.84 (0.81, 0.87)	0.58 (0.56, 0.62)
Skin and Subcutaneous (L00-L99)	1.10 (1.06, 1.14)	1.01 (0.97, 1.05)	0.77 (0.74, 0.80)	0.60 (0.57, 0.63)

Supplementary Table S6. Sensitivity Analysis Results: Incidence Rate Ratio (Median) and 95% sample interval of select Subchapters of disease, March 2020 to December 2020

Subchapter	Mar	Apr	May	Jun	Jul	Aug	Sept	Oct	Nov	Dec
I&P (J09-J18)	0.74 (0.71, 0.79)	0.30 (0.28, 0.33)	0.37 (0.34, 0.40)	0.59 (0.53, 0.66)	0.75 (0.68, 0.83)	0.68 (0.62, 0.76)	0.51 (0.46, 0.58)	0.35 (0.32, 0.40)	0.24 (0.21, 0.28)	0.18 (0.16, 0.21)
Acute URI (J00 – J06)	1.10 (1.05, 1.16)	0.34 (0.31, 0.37)	0.28 (0.26, 0.31)	0.39 (0.36, 0.44)	0.58 (0.53, 0.64)	0.51 (0.46, 0.57)	0.51 (0.46, 0.58)	0.52 (0.47, 0.59)	0.46 (0.42, 0.52)	0.35 (0.31, 0.40)
Acute LRI (J20-J22)	0.91 (0.86, 0.97)	0.26 (0.24, 0.30)	0.20 (0.19, 0.22)	0.24 (0.21, 0.27)	0.33 (0.30, 0.37)	0.31 (0.28, 0.35)	0.29 (0.26, 0.33)	0.31 (0.28, 0.36)	0.24 (0.22, 0.28)	0.16 (0.14, 0.18)
Viral skin and mucous (B00 – B09)	0.74 (0.70, 0.79)	0.34 (0.31, 0.38)	0.42 (0.39, 0.47)	0.44 (0.40, 0.49)	0.39 (0.35, 0.44)	0.43 (0.39, 0.48)	0.45 (0.41, 0.51)	0.50 (0.45, 0.56)	0.52 (0.48, 0.58)	0.52 (0.48, 0.59)
Middle ear (H65 – H75)	0.65 (0.62, 0.71)	0.19 (0.17, 0.21)	0.30 (0.28, 0.33)	0.41 (0.38, 0.46)	0.54 (0.49, 0.59)	0.59 (0.52, 0.67)	0.59 (0.51, 0.67)	0.50 (0.44, 0.58)	0.40 (0.35, 0.48)	0.32 (0.28, 0.37)
Intestinal Infectious (A00-A09)	0.68 (0.65, 0.72)	0.35 (0.32, 0.39)	0.42 (0.39, 0.45)	0.47 (0.43, 0.51)	0.52 (0.47, 0.56)	0.53 (0.48, 0.58)	0.57 (0.52, 0.63)	0.51 (0.46, 0.57)	0.44 (0.40, 0.51)	0.38 (0.34, 0.43)
Intellectual Disabilities (F70 – F79)	0.49 (0.42, 0.53)	1.77 (1.44, 1.91)	1.07 (0.82, 1.14)	2.11 (1.50, 2.29)	1.33 (0.89, 1.42)	1.24 (0.78, 1.33)	1.11 (0.68, 1.21)	1.12 (0.68, 1.24)	1.25 (0.76, 1.40)	1.21 (0.76, 1.34)
Schizophrenia (F20 – F29)	0.88 (0.75, 0.93)	1.23 (1.00, 1.33)	1.18 (0.95, 1.28)	1.24 (1.00, 1.32)	1.19 (0.94, 1.27)	1.20 (0.94, 1.28)	1.27 (0.99, 1.36)	1.23 (0.95, 1.33)	1.08 (0.87, 1.17)	1.13 (0.90, 1.23)
STI (A50 – A64)	0.81 (0.75, 0.90)	0.59 (0.52, 0.66)	0.75 (0.69, 0.86)	0.94 (0.87, 1.06)	1.10 (1.00, 1.23)	1.30 (1.19, 1.46)	1.25 (1.12, 1.41)	1.35 (1.21, 1.56)	1.14 (1.01, 1.34)	1.21 (1.10, 1.44)
Hemolytic anemias (D55 – D59)	0.74 (0.67, 0.79)	0.77 (0.68, 0.84)	0.98 (0.86, 1.06)	1.03 (0.88, 1.16)	1.26 (1.03, 1.37)	1.40 (1.10, 1.60)	1.52 (1.18, 1.79)	1.52 (1.24, 1.74)	1.23 (1.02, 1.46)	1.24 (1.05, 1.39)
