# Supplementary material for: Trends in non-COVID-19 hospitalizations prior to and during the COVID-19 pandemic period, United States, 2017–2021
Source: Nat Commun. 2022 Oct 8;13:5930. doi: 10.1038/s41467-022-33686-y (PMC9546751; doi:10.1038/s41467-022-33686-y)
Supplement: Supplementary file 3 — Reporting Summary [file 41467_2022_33686_MOESM3_ESM.pdf]

nature portfolio

Corresponding author(s): Kelsie Cassell

Last updated by author(s): 9/20/2022

Reporting Summary

Nature Portfolio wishes to improve the reproducibility of the work that we publish. This form provides structure for consistency and transparency in reporting. For further information on Nature Portfolio policies, see our Editorial Policies and the Editorial Policy Checklist.

Statistics

For all statistical analyses, confirm that the following items are present in the figure legend, table legend, main text, or Methods section.

n/a	Confirmed	
☐	☒	The exact sample size (*n*) for each experimental group/condition, given as a discrete number and unit of measurement
☒	☐	A statement on whether measurements were taken from distinct samples or whether the same sample was measured repeatedly
☐	☒	The statistical test(s) used AND whether they are one- or two-sided<br>*Only common tests should be described solely by name; describe more complex techniques in the Methods section.*
☒	☐	A description of all covariates tested
☐	☒	A description of any assumptions or corrections, such as tests of normality and adjustment for multiple comparisons
☐	☒	A full description of the statistical parameters including central tendency (e.g. means) or other basic estimates (e.g. regression coefficient) AND variation (e.g. standard deviation) or associated estimates of uncertainty (e.g. confidence intervals)
☒	☐	For null hypothesis testing, the test statistic (e.g. *F*, *t*, *r*) with confidence intervals, effect sizes, degrees of freedom and *P* value noted<br>*Give P values as exact values whenever suitable.*
☒	☐	For Bayesian analysis, information on the choice of priors and Markov chain Monte Carlo settings
☐	☒	For hierarchical and complex designs, identification of the appropriate level for tests and full reporting of outcomes
☒	☐	Estimates of effect sizes (e.g. Cohen's *d*, Pearson's *r*), indicating how they were calculated

Our web collection on statistics for biologists contains articles on many of the points above.

Software and code

Policy information about availability of computer code

Data collection	No software was used for data collection.
Data analysis	Data queries and analyses were run in SQL and R version 4.0.2 using the packages cluster-2.1.0, data.table-1.13.0, DBI-1.1.0, dplyr-1.0.2, ggplot2-3.3.2, glue, gridExtra-2.3, lubridate-1.7.9, patchwork-1.0.1, readr-1.3.1, stringr-1.4.0.

For manuscripts utilizing custom algorithms or software that are central to the research but not yet described in published literature, software must be made available to editors and reviewers. We strongly encourage code deposition in a community repository (e.g. GitHub). See the Nature Portfolio guidelines for submitting code & software for further information.

Data

Policy information about availability of data

All manuscripts must include a data availability statement. This statement should provide the following information, where applicable:

- Accession codes, unique identifiers, or web links for publicly available datasets
- A description of any restrictions on data availability
- For clinical datasets or third party data, please ensure that the statement adheres to our policy

The data that support the findings of this study are subject to controlled access due to the inclusion of protected health information in the dataset and restrictions on use set by COVID-19 Research Database consortium. Access to data is permitted upon application for use to the database and approval, via

contact@covid19researchdatabase.org. Responses to data requests are expected to take 3 months and access is limited to academic, medical, and scientific research of COVID-19 only.

Human research participants

Policy information about studies involving human research participants and Sex and Gender in Research.

Reporting on sex and gender	N/A
Population characteristics	N/A
Recruitment	N/A
Ethics oversight	Georgetown University IRB

Note that full information on the approval of the study protocol must also be provided in the manuscript.

Field-specific reporting

Please select the one below that is the best fit for your research. If you are not sure, read the appropriate sections before making your selection.

☐ Life sciences ☐ Behavioural & social sciences ☒ Ecological, evolutionary & environmental sciences

For a reference copy of the document with all sections, see nature.com/documents/nr-reporting-summary-flat.pdf

Ecological, evolutionary & environmental sciences study design

All studies must disclose on these points even when the disclosure is negative.

Study description	This study examined the temporal trends in diagnoses (ICD-10 Chapter and Subchapter) from Jan 1 2017 to June 1 2021. The aim of this analysis was to provide a monthly estimate of the incidence rate ratio (IRR), defined as the observed number of monthly hospitalizations divided by expected monthly hospitalizations, for each diagnostic chapter and subchapter of interest. Estimates were generated using Poisson regression models fit to the monthly count of diagnostic codes within each ICD-10 chapter (or subchapter) from the pre-pandemic period (January 1, 2017- December 31, 2019) to estimate the number of hospitalizations per month had the pandemic not occurred. Time series for each chapter and subchapter were analyzed independently. All models adjusted for time trends (linear trend) and yearly seasonality (using sine and cosine harmonic terms). While we did not have a true population denominator to use as an offset term in the model, due to a lack of information on payer population or hospital catchment areas, we estimated the size of the active patient population as the number of unique individuals with a recorded hospitalization per year for any cause. For the hospitalizations occurring between 2017 and 2019, the offset was the total number of unique individuals present in the same calendar year. Because there was a generally increasing trend in the number of individuals included in this database, the offset for 2020 and 2021 was estimated by fitting linear and seasonal trends to the monthly counts of unique individuals in the study prior to March 2020 and extrapolating these trends to the period from March 2020-June 2021. Prediction intervals for the final IRR estimates were calculated for each time point through a two-stage simulation using Monte Carlo resampling that accounted for parameter uncertainty and observation uncertainty.<br>Due to the numerous subchapters included in this analysis (N = 180), we implemented a hierarchical clustering algorithm (applying Ward's minimum variance method) to systematically group diagnoses that shared temporal trends in IRRs during the period between Jan 2020 and Jun 2021. The optimal number of clusters (k) was chosen via the "elbow method". This method attempts to maximize the variance (within-cluster sum of squares) explained by the clustering and minimize the number of clusters thereby excluding an increasing number of clusters that only provide a marginal gain in explained variance.
Research sample	The research sample included all patients reported to the database during the study period that did not meet exclusion criteria. The data collected by C19RDB is meant to represent insured (public and private) patients who were hospitalized within the United States.
Sampling strategy	No sampling or sample size calculation was used in this study as all hospitalizations that meant inclusion criteria were included for analysis. No subsamples of data were used.
Data collection	The data were collected through the COVID-19 Research Database consortium (Office_Ally project) through access to hospitalization billing clearing house information. This data was originally collected through electronic billing records.
Timing and spatial scale	The data were provided at the monthly scale for the time period between Jan 1, 2017 and Jun 1, 2021 because this was the smallest time scale permitted to report when using diagnostic code categories for analysis. Data were reported from all US states however this information was not included in our analysis since not all hospitals/healthcare centers could be accurately matched to a specific state or site.
Data exclusions	Exclusion criteria: Outpatient visits, hospitalizations that lacked a corresponding procedure code, hospitalizations for diagnoses that occurred fewer than 5000 times during the study period. See supplementary Figure S1 for N excluded.
Reproducibility	COVID-19 Research Database has prohibited the public sharing of code that was used to clean or generate the analysis used in this

Reproducibility	study for confidentiality reasons. COVID-19 Research Database states: "[The] database is housed within a secure Amazon Workspace (AWS) Virtual Environment. All analyses are conducted within the AWS environment. To ensure the security and privacy of the data, no code or data can be brought into the environment and no code can leave the environment."
Randomization	Randomization was not applicable to this study because we utilized all data available (that met inclusion criteria) in the analysis and this information was not prospectively collected as part of a clinical trial or similar study design.
Blinding	Blinding was not relevant because this was an ecological analysis without intervention. All billing data (used for analysis of diagnostic codes) was anonymized (random ID number assigned) so that researchers did not require blinding and were interested in aggregate trends rather than individual patient level trends.

Did the study involve field work? ☐ Yes ☒ No

Reporting for specific materials, systems and methods

We require information from authors about some types of materials, experimental systems and methods used in many studies. Here, indicate whether each material, system or method listed is relevant to your study. If you are not sure if a list item applies to your research, read the appropriate section before selecting a response.

Materials & experimental systems

n/a	Involved in the study	
☒	☐	Antibodies
☒	☐	Eukaryotic cell lines
☒	☐	Palaeontology and archaeology
☒	☐	Animals and other organisms
☒	☐	Clinical data
☒	☐	Dual use research of concern

Methods

n/a	Involved in the study	
☒	☐	ChIP-seq
☒	☐	Flow cytometry
☒	☐	MRI-based neuroimaging
